# Practitioner perspectives on symptomatic faecal immunochemical testing in the UK: a qualitative interview study

**DOI:** 10.3399/BJGP.2024.0358

**Published:** 2025-05-07

**Authors:** Christina Dobson, Adam Biran, Colin Rees, Willie Hamilton, Christian von Wagner, John Whelpton, Linda Sharp

**Affiliations:** Population Health Sciences Institute, Newcastle University, Newcastle upon Tyne.; Population Health Sciences Institute, Newcastle University, Newcastle upon Tyne.; Population Health Sciences Institute, Newcastle University, Newcastle upon Tyne, and British Society of Gastroenterology, London.; University of Exeter Medical School, Exeter.; Institute of Epidemiology and Health Care, University College London, London.; Population Health Sciences Institute, Newcastle University, Newcastle upon Tyne.

**Keywords:** bowel symptoms, colorectal cancer, faecal immunochemical test, healthcare professionals, primary health care, qualitative interview study

## Abstract

**Background:**

Faecal immunochemical testing (FIT) is now core to the management of patients presenting in primary care with symptoms of possible colorectal cancer. Patients with a positive FIT (≥10 μg haemoglobin [Hb]/g faeces) qualify for an urgent suspected cancer referral. FIT-negative patients are typically managed in primary care or referred through routine pathways.

**Aim:**

To examine practitioners’ experiences of delivering symptomatic FIT, identifying perceived benefits, disbenefits, and implementation issues, to inform potential future service improvements.

**Design and setting:**

A qualitative interview study with primary and secondary care health professionals involved in delivering symptomatic FIT pathways from across the UK.

**Method:**

Thirty semi-structured interviews were conducted with professionals from a range of specialties. An iterative topic guide informed interviews while allowing freedom to explore novel lines of enquiry. Pseudo-anonymised transcripts were coded, and themes were identified and developed.

**Results:**

Symptomatic FIT was seen to be beneficial for increasing confidence in clinical decision making and enriching the pool of patients being definitively investigated for colorectal neoplasia. There were varying views on the impact of symptomatic FIT on workload with the burden of additional workload generally seen to impact primary care. Concerns about current practice included overuse of FIT, burden of investigations in patients with false-positive results, and diagnostic delays for both cancer and benign disease. Uncertainties existed around management of patients with rectal bleeding, appropriate strategies for safety netting, and the value of repeat FIT.

**Conclusion:**

Symptomatic FIT is largely seen as beneficial; however, health professionals would welcome further evidence and guidance around optimal application.

## Introduction

Colorectal cancer (CRC) is the fourth most commonly diagnosed cancer in the UK and second most common cause of cancer death, responsible for more than 42 000 new cases, and more than 16 000 deaths each year.^1^ Outcomes are better when patients are diagnosed with early-stage disease, but the majority of CRCs in the UK are diagnosed at an advanced stage (III and IV); 5-year survival for CRC in the UK is improving but remains lower than in several comparable countries.^2^

Early diagnosis has been at the core of consecutive cancer strategies in the UK,^3^ leading to the introduction of national Bowel Cancer Screening Programmes,^4^ public health awareness campaigns,^5^ and National Institute for Health and Care Excellence (NICE) referral guidelines.^6^ These initiatives have not only expedited investigations but have also placed unprecedented demand on endoscopy services.^7^ These demands were further exacerbated by the COVID-19 pandemic^8^ when roll-out of faecal immunochemical testing (FIT) for triage of symptomatic individuals was accelerated.^9^ It was anticipated that so-called ‘symptomatic FIT’ would simultaneously help manage endoscopy demand, ensure prompt investigation of those at greatest risk of CRC, and avoid unnecessary procedures for those at lowest risk.

Previously, patients presenting in primary care were referred for further investigation based on assessment of symptoms. FIT is now used to inform decisions on the necessity and urgency of definitive investigation in adults, alongside symptom information. Patients are required to collect a sample of their stool using a dedicated kit and send the sample to a laboratory for analysis. FIT detects and quantifies haemoglobin (Hb) within the stool. For symptomatic patients, a concentration of ≥10 μg Hb/g faeces is considered a ‘positive’ result.^10^ These patients qualify for an urgent suspected cancer referral (previously called the ‘2-week-wait’ pathway) for investigation of their symptoms, primarily by colonoscopy, or computed tomography colonography (CTC) in those for whom a colonoscopy is inappropriate.^6^

**Table:** How this fits in

In the UK, faecal immunochemical testing (FIT) is now central to referral for definitive investigation of patients presenting in primary care with symptoms of possible colorectal cancer. This is one of the first studies to explore implementation of symptomatic FIT from a range of health professional perspectives. Symptomatic FIT is viewed positively by clinicians, particularly as a tool to support clinical decision making. However, there remain areas of uncertainty that require further evidence and guidance, including safety-netting strategies, the role of repeat FIT, and utilisation in patients with active rectal bleeding.

The aim of this qualitative study was to explore health professionals’ experiences of delivering symptomatic FIT to learn from early implementation, identify potential areas for improvement, and inform future service development. The study formed part of a wider programme of work examining symptomatic FIT that, importantly, also explored patient experiences and perspectives.^11^ The patient findings will be presented in a subsequent paper.

## Method

### Recruitment and sampling

Health professionals involved in delivering symptomatic FIT were identified through NHS hospital sites (North East, Yorkshire, and Midlands), membership of the British Society of Gastroenterology (BSG)/Association of Coloproctology of Great Britain and Ireland (ACPGBI) symptomatic FIT guideline development group, and snowball sampling. This approach was intended to recruit individuals differing in expertise and experience of symptomatic FIT. Identified individuals were emailed an invitation to participate. Willing participants contacted the research team directly. Participants were purposively sampled by specialty, and diversity was sought in relation to gender, region, and experience of symptomatic FIT delivery (for example, guidelines group involvement) to ensure we engaged with a range of experiences and perspectives (Table 1).

**Table 1.** Interview participants

**Participant ID**	**Specialty**	**Gender**	**Recruitment**
P001	GP	Female	Guidelines group
P002	GP	Female	Snowball sampling
P003	GP	Male	Snowball sampling
P004	GP	Female	Snowball sampling
P005	GP	Female	Snowball sampling
P006	GP	Male	Snowball sampling
P007	GP	Female	Snowball sampling
P008	Gastroenterologist	Male	Guidelines group
P009	Gastroenterologist	Male	Guidelines group
P010	Gastroenterologist	Male	Guidelines group
P011	Gastroenterologist	Male	Snowball sampling
P012	Gastroenterologist	Male	Snowball sampling
P013	Gastroenterologist	Female	North East Hospital
P014	Gastroenterologist	Male	North East Hospital
P015	Surgeon	Male	Guidelines group
P016	Surgeon	Male	Guidelines group
P017	Surgeon	Male	Guidelines group
P018	Surgeon	Female	Midlands Hospital
P019	Surgeon	Female	Midlands Hospital
P020	Surgeon	Male	Yorkshire Hospital
P021	Gastroenterology Nurse Coordinator	Female	North East Hospital
P022	Consultant Nurse	Female	Snowball sampling
P023	Nurse Endoscopist	Female	Guidelines group
P024	Nurse Endoscopist	Female	Yorkshire Hospital
P025	Nurse Practitioner	Female	Midlands Hospital
P026	Inflammatory Bowel Disease Nurse	Female	Yorkshire Hospital
P027	Colorectal Cancer and Stoma Nurse	Female	North East Hospital
P028	Pathology Lab Director	Female	Snowball sampling
P029	Consultant Biochemist	Male	Guidelines group
P030	Radiologist	Male	Guidelines group

### Data collection

Semi-structured interviews were conducted remotely on Microsoft Teams or by telephone between September 2022 and June 2023. Interviews were conducted with one of two experienced qualitative health researchers with expertise in colorectal cancer pathways and non-clinical backgrounds. Consenting occurred immediately prior to interview and was audio-recorded. A topic guide, developed by clinical and non-clinical research team members (including a lay co-author) and reviewed by our Patient and Public Involvement (PPI) panel, was used across interviews (Supplementary Information S1). Topics included experience of FIT, implementation of FIT, FIT application in different patient groups, and perceived advantages and challenges. The topic guide was applied flexibly and iterated, allowing novel areas to be pursued within interviews and with subsequent interviewees. Interviews were audio-recorded and lasted 20–60 minutes (median length of 39 minutes). Recordings were pseudo-anonymised, professionally transcribed, and checked for transcription accuracy by the research team. Recruitment ceased when interviews repeatedly supported emerging themes and a point of acceptable accuracy was believed to have been reached within the data.^12^

### Data analysis

Initial codes were developed from the topic guide and insights from researchers’ notes. Codes were independently applied to three transcripts by two co-authors and were discussed and revised. The revised code list was applied to all transcripts; however, analysis remained open to the identification of new codes and topics, which were incorporated and applied to earlier transcripts. Coding occurred concurrently with fieldwork, to guide recruitment. NVivo 13 software was used to organise coded data and aid collaborative analysis. Inductive thematic analysis^13^ was used to map out significant changes and consequences arising from symptomatic FIT implementation, as well as uncertainties, and perceived challenges for future delivery. Themes were discussed and refined across the wider research team, including a lay co-author, with findings and interpretation also shared with our PPI panel for sense checking.

## Results

We interviewed 30 health professionals involved in delivery of symptomatic FIT (Table 1).

### Findings

We organised data around two overarching themes: ‘Current impacts’ (with subthemes of workload, primary care decision making, and seeing the ‘right’ patients at the ‘right’ time); and ‘Areas of uncertainty’ (with subthemes of rectal bleeding, safety netting, repeat FIT, and positive result thresholds).

### Current impacts

#### Workload.

Participants described changes to workload, particularly the administrative burden associated with symptomatic FIT. These changes were experienced differently by health professionals and administrative staff, and across primary and secondary care:

*‘Making sure everyone has to have a FIT test beforehand effectively puts increasing pressure on primary care.’*

(P001, GP, female [F])

*‘The amount of active triage that has gone on … that does carry a burden of admin time.’*

(P008, Gastroenterologist, male [M])

Implementing symptomatic FIT in primary care entailed additional workload associated with ordering tests and obtaining results, but most notably from following up patients who had not returned their test:

*‘It creates work for our admin staff, because I’m then messaging them going “I haven’t got this FIT test result back. Can you ring the patient and see if he’s willing to do the FIT test? So, can we re-request it?”’*

(P007, GP, F)

Additional primary care workload was also perceived by some to have arisen from the management of FIT-negative patients, who may have previously been referred for further investigation. By contrast, others acknowledged that a negative result could reduce the need for ‘watchful waiting’, saving time associated with follow-up consultations:

*‘Primary care physicians* [used to] *see a patient when they have those symptoms … refer them and that’s job done … In the new pathway … GPs will have to wait for the results … Then take a decision. Then review the patient again if the patient’s negative … the work implication of that is significant.’*

(P011, Gastroenterologist, M)

*‘I don’t think there’s any significant additional workload from doing the test or from interpreting the results … overall workload … is reduced … because when you’re using time as your diagnostic tool you need further consultations … that saving in time is probably the most significant element.’*

(P006, GP, M)

In secondary care there were mixed views on the impact of symptomatic FIT on numbers of definitive investigations performed:

*‘The numbers going to colonoscopy have fallen by about half from this pathway.’*

(P008, Gastroenterologist, M)

*‘We’ve noticed a significant reduction in the number of CT colonograms we’re doing for this diagnostic population.’*

(P030, Radiologist, M)

*‘I think there’s been an increase in referrals.’*

(P023, Nurse Endoscopist, F)

*‘I don’t know if the number of referrals has impacted, because it’s been so variable.’*

(P012, Gastroenterologist, M)

The relative burden of additional work associated with symptomatic FIT was identified by some as primarily impacting primary care:

*‘It’s then putting it on us to chase it, so it’s another appointment sometimes, so you’re doubling workload. Instead of saying “Look, FIT test done, let the hospital chase it.” Why do we have to chase it? Because they’ll still look and triage the thing, it’s about targets for them.’*

(P004, GP, F)

Minimising additional GP workload was seen as important to the success of symptomatic FIT. Proposed solutions included provision of additional resources at the primary care network (PCN) level, and additional specialist nurse time in secondary care for triage:

*‘If there could be a mechanism by which we can have a person who is responsible at a PCN level to chase the patient who is missed out, to chase the patient with the results, ensure that that pathway is there. We need to reduce the workload because they* [GPs] *are already overworked.’*

(P011, Gastroenterologist, M)

*‘Due to securing some extra funding for extra nurse specialists to do the triage step, we’ve agreed in the short-term to trial all patients being referred at the point they send the FIT off. So, the FIT and the referral will then be acted upon in secondary care by the triage step.’*

(P030, Radiologist, M)

#### Primary care decision making.

FIT was welcomed by many GPs, especially those with less experience, as a means to increase confidence in clinical decision making and manage risk:

*‘It’s a really good tool to make a decision, especially for relatively inexperienced GPs. I’m not maybe as accustomed to kind of taking on risk as a more senior clinician. So, for someone like me, your juniors, I think it’s a really good tool to add a bit of information and weighting to a decision you’re making.’*

(P003, GP, M)

#### Seeing the ‘right’ patients at the ‘right’ time.

In secondary care time spent delivering symptomatic FIT was seen as a useful investment; it was perceived to target investigations at those most at risk, and move colonoscopy away from a diagnostic tool, towards a primarily therapeutic one:

*‘FIT has allowed us to investigate the right patients, the more higher-risk patients and try and meaningfully distribute our diagnostics.’*

(P022, Consultant Nurse, F)

*‘I’d much rather … have colonoscopy moving in the direction of ERCP* [endoscopic retrograde cholangiopancreatography] *and be a therapeutic test, rather than a diagnostic test.’*

(P008, Gastroenterologist, M)

However, participants felt that the anticipated benefits (reducing endoscopy burden or increasing cancer yield) were not necessarily realised. Possible explanations included overuse of FIT by GPs driving unnecessary investigation of patients with false-positive FIT results. There was also acknowledgment that concerns about potential for legal reprisals may drive overuse of FIT:

*‘The referrals apparently have gone up but the cancers that we’re picking up have not gone up.’*

(P027, Colorectal Cancer and Stoma Nurse, F)

*‘We were hoping that FIT would reduce the amount of fast-track investigations that we were doing, but actually it’s had the opposite effect … GPs seem to have latched on to doing FITs for all sorts of indications and, of course, some of those FITs are going to turn up positive … so we’re getting referred patients with slightly positive FITs for colonoscopy as a fast track, when previously they wouldn’t have even been referred.’*

(P020, Surgeon, M)

*‘There’s a fear of medicolegal reprisal if they* [GPs] *miss a cancer.’*

(P010, Gastroenterologist, M)

There were also concerns that negative FIT results could ‘downgrade’ the importance of symptoms and introduce delays in the diagnosis and treatment of non-cancer conditions:

*‘A lot more people are sort of going through the process because they’re FIT negative, discharged, but then promptly being re-referred back because the GPs in primary care want advice on management. So, I think with our current process it’s actually prolonging patient pathways.’*

(P009, Gastroenterologist, M)

*‘I think there’s big concerns about downgrading all these patients … they may have very severe symptoms still, but just because their FIT test is negative, they get downgraded … so they take two years to see them. I think we all share that concern. It’s just moving a problem to elsewhere really.’*

(P015, Surgeon, M)

This participant also reflected that symptomatic FIT had reduced the number of low-risk patients on the suspected cancer pathway, which allowed more time to see patients with non-cancer conditions:

*‘It frees a bit more space in outpatients to see patients with other conditions, more equally needy benign conditions, such as inflammatory bowel disease.’*

(P015, Surgeon, M)

Even for patients with positive FIT results, there were concerns about timeliness of investigation, because of perceived delays arising from requesting, processing, and reporting FIT prior to referral, although the clinical significance of such delays was questioned:

*‘If I examine someone who has a suspected mass in the abdomen and has had weight loss, the* [DG30] *NICE guidelines would say we can refer* [urgently]*. The FIT testing protocol currently would say I need to give the FIT test to the patient to do the FIT test, they need to return that test; if they don’t return it, I need to chase that test. All of that delays the two-week-wait referral.’*

(P001, GP, F)

*‘It adds a little extra work in doing the test, interpreting it, delaying it. It means there’s sometimes a short delay for patients in getting the referral but it’s unlikely to be of any clinical significance.’*

(P006, GP, M)

### Areas of uncertainty

#### Rectal bleeding.

Participants expressed uncertainty and mixed opinions as to the appropriateness of FIT in patients with active rectal bleeding:

*‘If somebody’s had like a haemorrhoidal bleed, it’s inevitable that we’re going to get a result above four hundred* [µg Hb/g faeces]*. And I think those are sometimes, in my opinion, the least likely to find pathology.’*

(P024, Nurse Endoscopist, F)

*‘*[The GP] *might have got in a history, some bleeding from like an innocent pile or something, they’re given a FIT test, but there’s probably going to be blood in that and then they end up with like a two-week-wait colonoscopy.’*

(P003, GP, M)

#### Safety netting.

Safety netting of patients with a negative FIT result was frequently raised, particularly in the context of worsening symptoms, or a family history of cancer:

*‘I don’t think you have a robust system that you go, “oh your FIT’s less than ten* [µg Hb/g faeces]*, we’re never going to see you on a cancer pathway”. You turn round and go “actually, this patient’s symptoms are getting worse, it’s becoming a bit more alarming. Yes, their FIT was negative, but let’s either do a process where they have another FIT, or actually they go for a colonoscopy” and allow our primary care colleagues, who are phenomenal diagnosticians and clinicians, to use their experience and knowledge.’*

(P030, Radiologist, M)

*‘You can combine a FIT and family history risk, but it would be a little bit of a step backwards to just work with FIT for them, because so much work has already been done on assessing their risk because of their family history.’*

(P023, Nurse Endoscopist, F)

The possibility of missing a cancer at a site beyond the colon was also a concern. Some participants discussed using other tests alongside FIT to improve cancer detection and diagnosis of other conditions:

*‘There’s a relatively high incidence of upper GI cancers and pancreatic cancers that are detected in the course of patients having other investigations alongside a FIT test … there has to be something built in to pick up other cancers that wouldn’t necessarily cause a high FIT but can produce the same type of symptoms … I think the evidence needs to come along and become more mature in the role of how other blood tests would fit into that whole pathway … whether there’s a role for one of these multi-cancer detection tests, either alongside, or potentially instead of, FIT.’*

(P015, Surgeon, M)

#### Repeat FIT.

There was uncertainty as to when, and for whom, repeating a FIT would be appropriate, but suggestions that this could play a role in safety netting of some groups of patients, such as those with rectal bleeding, or ‘borderline’ faecal Hb concentrations:

*‘Where you’ve got a negative FIT, you do as a backstop, second FIT at a later stage to ensure that it’s a correct number. I don’t see any major issues with that. I think it needs to be evidence-based if it’s going to be implemented.’*

(P030, Radiologist, M)

*‘There’s the other question of whether a second FIT test can be done as a part of your safety-netting work … I think that is a reasonable thing to do and that is at the point of your six-week review for patients who’re FIT negative, and the patient’s symptoms have persisted, I think there is no harm in doing a second FIT test at that time.’*

(P011, Gastroenterologist, M)

Several participants wanted more evidence and guidance on the application of repeat FIT, in relation to both patient groups, and clinically appropriate intervals between tests:

*‘I don’t think the evidence base is, there’s not much around this, but I don’t think they should be on the same day, I think they should at least be on different days maybe and I think a study would be needed to determine that.’*

(P026, Inflammatory Bowel Disease Nurse, F)

*‘If the patient’s reluctant to have tests because their symptoms are settling down, but the FIT is perhaps eleven or twelve* [µg Hb/g faeces]*, so just over the border of positive. We might say, “well let’s repeat the FIT in say six weeks’ time and if it’s negative then you don’t need any tests, if it’s positive then maybe we should be taking a look.”’*

(P022, Consultant Nurse, F)

#### Positive result threshold.

Several participants discussed the threshold for a positive FIT result, in relation to concerns about missed cancers and the lack of differentiation between mildly and substantially raised Hb concentrations. Some had confidence that the current threshold was set appropriately by people with expertise; others suggested it was arbitrary or wanted to see the evidence base:

*‘We’re using a cut-off which is an arbitrary cut-off based on a consensus statement not on firm evidence.’*

(P001, GP, F)

*‘There’s a big difference, for me, between a patient who has had a FIT done for very iffy indications and it’s fifteen* [µg Hb/g faeces]*, which technically is raised, and then the patient who has suspicious symptoms and a FIT of over four hundred* [µg Hb/g faeces]*, yet the system we have lumps those patients all in together.’*

(P020, Surgeon, M)

## Discussion

### Summary

This study explored health professionals’ experiences of implementing symptomatic FIT. Practitioners’ perspectives are particularly valuable as they will ultimately determine how consistently guidance and referral pathways are followed, which, in turn, has implications for the NHS and patients.

Symptomatic FIT was generally received positively and was seen to offer advantages, including improved confidence in clinical decision making and an enriched pool of patients undergoing colonoscopy for suspected colorectal neoplasia. However, perceptions of symptomatic FIT were sometimes contradictory and not always positive. Participants’ concerns included potential overuse of FIT in primary care and the impact of this on referral numbers. Some also raised the issue of increased primary care workload associated with overseeing FIT requests and completion, and managing patients with negative results. Concerns about possible diagnostic delays were raised for both cancer and non-cancerous disease. Although some secondary care clinicians reported that FIT-based triage freed up more time to see patients with benign conditions, lengthy wait times for routine referrals remained a concern.

Participants expressed uncertainty around the effectiveness and applicability of FIT for certain patient groups, including those with active rectal bleeding, a family history of cancer, or another underlying cancer. There were calls for further evidence and guidance around the application of repeat FIT testing. Some participants expressed discomfort at the lack of discrimination in current pathways between mildly and extremely elevated faecal Hb concentrations.

### Strengths and limitations

The inclusion of health professionals delivering different elements of symptomatic FIT pathways allowed comprehensive examination of experiences and challenges. However, specialties were unevenly represented in the sample, as were geographic locations; although there were no restrictions placed on location, particularly with respect to recruitment of guidelines group members, only three NHS Trusts (albeit in different Cancer Alliance areas) were involved in active recruitment. It is worth noting that, while cancer referral guidelines vary across nations, the BSG/ACPGBI guidance applies across the UK and, although there may be degrees of variation in local implementation (because of, for example, variations in workload, workforce availability, and/or case mix), most findings will likely have considerable transferability.

Involvement of a PPI panel and lay co-author in reviewing data collection tools and sense checking the study findings and interpretations was a key strength. The focus of these data was practitioner experiences of implementation; however, ensuring that patient perspectives were incorporated into interpretation of results added rigour and increased broader relevance of the analysis and reporting.

Interviews took place between September 2022 and June 2023, which is 2–3 years after widespread adoption of symptomatic FIT occurred around mid–2020. Perceptions of FIT may have changed over time as individuals and systems adapted, as well as in response to the NICE Diagnostic Guideline (DG) 56 for use in England,^6^ which was published after data collection.

There is potential self-selection bias in the sample: those with strong opinions (positive or negative) about symptomatic FIT may have been more likely to take part. We sought to minimise the impact of this by recruiting through multiple routes. Finally, the study was not designed to compare perspectives across different professional (sub)groups.

### Comparison with existing literature

Concerns about symptomatic FIT causing diagnostic delays have been previously raised.^14^ This study provides, to our knowledge, the first empirical data on this from practitioners. Additional time-to-diagnosis arising from symptomatic FIT will likely be clinically negligible for those who return FIT promptly, but for those who need multiple reminders, or who fail to return a test, it is possible that diagnostic intervals could be notably longer than in pre-FIT pathways. This in turn may impact stage distribution and outcomes, which in turn could exacerbate existing CRC inequalities, for instance, among those from areas of high deprivation^15^^,^^16^ or minority ethnic populations.^17^

**Figure 1.**
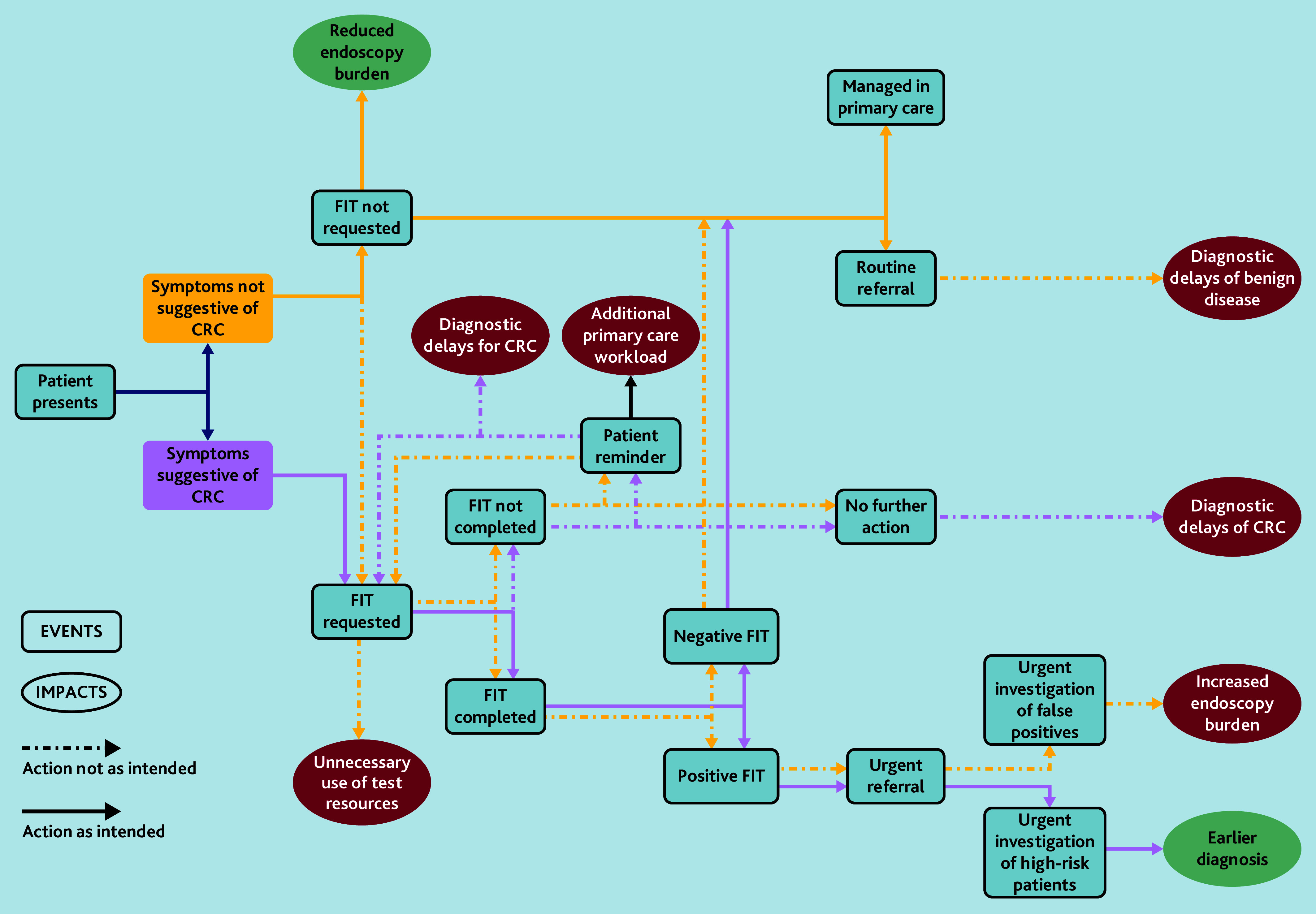
Model of symptomatic FIT pathway delivery and consequences. CRC = colorectal cancer. FIT = faecal immunochemical testing.

Introducing new ways of working can yield intended and unintended consequences that may impact stakeholders disproportionately. The importance of unintended consequences arising from changes to endoscopy services has been documented previously, including unintended negative impacts on patients.^18^^,^^19^ Examples of such consequences identified by participants in this study include overuse of FIT leading (some) GPs to over-refer patients; additional secondary care resourcing requirements to triage referrals; and the perceived introduction of diagnostic delays for those with non-cancerous conditions sharing symptoms with CRC (Figure 1).

Some previous work has suggested that dual/replicate FIT (that is, two simultaneous tests) increases sensitivity, reduces missed colorectal pathology, has an acceptable impact on workload, and is a superior predictive tool to single FIT.^20^^,^^21^ However, neither this evidence, nor that on the role of repeat FIT (that is, two tests with an interval between) is clear or consistent.^22^ The NICE guideline DG56 calls for more research into the application and predictive value of dual FIT,^6^ and this study highlights health professionals’ desire for further examination of the role of repeat FIT. Research into appropriate intervals between repeat FITs and the natural fluctuations in Hb concentrations over time would also be valuable.

Participants discussed uncertainty about the appropriateness of FIT in patients with rectal bleeding, although FIT has been shown to still be useful with these patients and is recommended for use in this patient group.^10^

### Implications for research and practice

This study highlights a number of practitioner perceptions of unintended consequences and disbenefits arising from the introduction of symptomatic FIT, as illustrated in Figure 1. Future monitoring and evaluation to capture and quantify these is essential to inform mitigation. Furthermore, prior to the introduction of significant changes to practice, greater effort should be made to consider potential adverse effects in detail, for instance, using dark logic models.^19^ Long-term implementation seems most likely to succeed if changes in practice are perceived as beneficial and equitable by those delivering the service. Monitoring diagnostic intervals for those diagnosed with cancer and non-cancerous conditions, and scrutinising cancer stage and outcomes, would allow possible negative impacts to be better assessed.

Further understanding of relative and absolute risk of colorectal neoplasia at different Hb concentrations is required, to ascertain the potential for further tailoring of pathways, and safety netting of patients with borderline FIT results.^23^^,^^24^ Such tailored pathways might also help address concerns around diagnostic delays, by expediting investigation for those at greatest risk even further. In relation to concerns about long wait times for specialist opinion in patients with non-cancer conditions, mechanisms to support patients awaiting specialist opinion for these conditions need to be considered, to manage symptoms in the interim, and expedite diagnosis.

Repeat FIT has potential to reduce over-referral, tailor pathways for those with mildly positive FIT results, as well as reduce missed CRC diagnoses.^20^ However, more evidence is needed to understand thresholds at which repeat FIT is valuable, appropriate intervals between tests, and fluctuations in faecal Hb concentrations over time, in both ‘healthy’ and ‘neoplastic’ populations.^22^

Although there is evidence supporting the use of FIT in patients with active rectal bleeding, given the uncertainty about use in this patient group, further (and clearer) communication of this evidence to primary care practitioners would be beneficial. Practitioners would welcome evidence-based guidance with regards to repeat testing and tailoring of FIT-positive pathways to expedite diagnosis.
