# Supplementary material for: Practitioner perspectives on symptomatic faecal immunochemical testing in the UK: a qualitative interview study
Source: Br J Gen Pract. 2025 May 7;75(755):e390–6. doi: 10.3399/BJGP.2024.0358 (PMC12070297; doi:10.3399/BJGP.2024.0358)
Supplement: Supplementary file 1 [file BJGP.2024.0358_suppl.pdf]

Supplementary Information S1: Interview Topic Guide: Health Care Practitioners

A: Intro and FIT Experience

- To begin with, please would you tell me what your professional role is.
- In your role, what contact do you have with patients presenting with symptoms of possible colorectal cancer?
- Have you been involved in using FIT-to triage patients with possible symptoms of colorectal cancer?
- *If no:*
    - *Do you envisage being involved in this in future?*
        - *In what way?*

If yes:

- When did you start using FIT for this purpose?

B: How FIT is used / specific activities / workload

- Can you describe how you would generally use FIT in the assessment and decision-making process for a typical symptomatic patient?
- How does this differ from your previous practice?
- Has the introduction of FIT-based triage changed your clinical practice?
  - In what ways?
- What specific actions or activities do you personally have to do to use FIT-based triage?
- Are you able to do these things effectively?
- What specific actions or activities do you need other people to do?
  - Are these activities performed effectively?
- Have you encountered any barriers to effectively delivering FIT-based triage?
- What impact has the introduction of FIT-based triage had on your personal workload?
- What impact has it had on the workload of your wider team?
- Has it changed the distribution of workload?
- Does your practice/clinical team undertake any monitoring, feedback, or discussion, around the use of FIT with symptomatic patients, or discussion of unusual cases?
- Do you think FIT is – or will be - easily integrated into the appraisal and management pathways for symptomatic patients?
  - Is there anything about the integration of FIT into patient pathways which you think will be challenging (either in terms of your own practice or more generally)?
  - Is there anything that needs to be put in place to help ensure that FIT-based triage works as well as possible?
- Have you encountered any problems using FIT with symptomatic patients, from either the health care delivery side, or from patients?
- Is there anything you think could be done to mitigate these problems?

C: Advantages / disadvantages and concerns

- What do you think about using FIT with symptomatic patients?
  - Are there any advantages?
    - For the health service?
    - For patients?
  - Are there any disadvantages?
    - For the health service?
    - For patients?
- Are there any patient groups you have concerns about in the context of FIT-based triage?
  - Who are they and why?
  - How do you think this patient group should best be managed?
- What do you do if a patient does not complete their FIT test?
- Are there contexts, or patient groups, for whom you think it might not be appropriate to request a FIT?
  - Who are they and why?
  - How would you manage this patient group?
- Are there contexts in which you would want to obtain repeat FIT scores to inform your decision-making?
  - What/who are they and why?
  - How many repeat scores would you want?
  - Over what time period?

D: Training / guidelines

- Have you received any training since you started using FIT to appraise symptomatic patients? If so, what training did you receive?
- Do you refer to any guidelines or protocols to guide your use of FIT-based triage of symptomatic patients?
    - What guidelines are they?
    - How useful are they (or not) and why?
    - Are there contexts in which you would deviate from the guidelines in your management of a patient?
    - ‘Is there anything that would be useful to support your use of FIT with symptomatic patients, for instance, professional training, prompts during consultations, [or guidelines *if not mentioned*]?

E: Patient interaction and communication

- What do patients tell you about their experiences of using the FIT kit at home?
- How are FIT results and their implications communicated to patients?
- How do you think patients understand the meaning of FIT results?
- What do you think patients understand about how these results are used for decision making?
- How acceptable do you think patients find this use of FIT?

For practitioners who have NOT delivered FIT triage

- What advantages do you think might come from FIT-based triage of symptomatic patients?
- What concerns would you have about FIT-based triage of symptomatic patients?
- Are there any contexts in which you imagine it might not be appropriate, or possible, to get a patient to provide a sample for FIT?
    - How do you think these patients should best be managed?

- What concerns would you have about integrating FIT into the appraisal and management pathways for symptomatic patients?

- Do you see any potential challenges in the introduction of FIT
    - with respect to your personal, clinical practice?
    - with respect to your wider team's practice?
    - for health care services more generally?
- Is there anything that would help you in your use of FIT with patients presenting with symptoms of possible colorectal cancer, for instance, professional training or prompts during consultation?
- Do you think that your patient population would be able, and willing, to complete FIT tests?
    - Do you anticipate there being reluctance to complete FIT from any particular patient groups?
        - Why do you think this might be?
        - What do you think could be done to mitigate this?
